# Histological evaluation of hysterectomy specimens after NovaSure^®^ endometrial ablation in patients with atypical endometrial hyperplasia or endometrial carcinoma

**DOI:** 10.1007/s00404-026-08497-x

**Published:** 2026-06-18

**Authors:** Oliver M. Schleicher, Alaa Hamzeh, Julia Gocke, Patrik Poeschke, Frederik A. Stuebs, Stefanie Burghaus, Felix Heindl, Arndt Hartmann, Matthias W. Beckmann, Christian Matek, Julius Emons

**Affiliations:** 1 Department of Gynecology and Obstetrics, Comprehensive Cancer Center Erlangen-EMN (CCC ER-EMN), Erlangen University Hospital, Friedrich-Alexander-Universität Erlangen-Nürnberg, Erlangen, Germany; 2 Institute of Pathology, Comprehensive Cancer Center Erlangen-European Metropolitan Area of Nuremberg (CCC ER-EMN), Erlangen University Hospital, Erlangen, Germany

**Keywords:** NovaSure® endometrial ablation, atypical endometrial hyperplasia, endometrial carcinoma

## Abstract

**Background:**

Treatment with NovaSure® endometrial ablation is approved for patients with heavy menstrual bleeding (HMB) without evidence of malignant or premalignant lesions. This analysis addresses the rare but clinically relevant situation in which endometrial carcinoma (EC) or atypical hyperplasia (AEH) is identified histologically after endometrial ablation in premenopausal patients.

**Objective:**

Histological evaluation of hysterectomy specimens with correlation to clinical parameters in patients undergoing hysterectomy after incidental histological diagnosis of AEH or EC following NovaSure® endometrial ablation.

**Methods:**

A retrospective single-center analysis was conducted on more than 400 patients who underwent NovaSure® endometrial ablation at our center between January 2020 and February 2025. Patients with AEH or EC for whom subsequent hysterectomy specimens were available were included. Histological evaluation was performed and independently reviewed to assess residual endometrium, residual endometrial atypia or carcinoma, and ablation-related histomorphological changes.

**Results:**

A total of 11 patients (AEH *n* = 8; EC *n* = 3) underwent subsequent hysterectomy after NovaSure® endometrial ablation. Six out of eight patients with AEH showed no residual atypia in the hysterectomy specimens (2/8 with focal residual atypia), and no residual invasive carcinoma was detected in any of the three carcinoma cases. Histopathological analysis showed pronounced postablative changes, including necrosis, fibrosis, zonation, and vascular and lymphatic alterations.

**Conclusion:**

This descriptive study provides a clinicopathological characterization of patients with EC or AEH who underwent hysterectomy after endometrial ablation. In these patients, no preprocedural evidence of endometrial pathology was present, and the diagnosis was established solely through routine histopathological examination of curettage specimens obtained immediately before the ablation procedure. Histological assessment revealed pronounced changes, highlighting specific diagnostic challenges and underscoring the importance of careful patient selection and thorough diagnostic evaluation before NovaSure® ablation. Within the limitations of this study, no evidence was found that prior endometrial ablation compromises oncological outcome.

## What does this study add to the clinical work?

**Table:** 

Even after a thorough preoperative evaluation, there are rare cases in which atypical endometrial hyperplasia or endometrial carcinoma are incidentally diagnosed following NovaSure® endometrial ablation. This study describes the histological changes observed after ablation and highlights relevant interdisciplinary diagnostic and therapeutic challenges.	

## Introduction

Abnormal uterine bleeding (AUB) affects approximately 10–20% of all women, and is defined as deviations from the normal menstrual pattern in terms of frequency, regularity, duration, or volume. Depending on the underlying cause, there are various conservative treatment options (e.g., hormonal) [1, 2]. In cases of failure, rejection, or inapplicability of conservative therapy, NovaSure® endometrial ablation is a less-invasive treatment option compared to hysterectomy in women with HMB. The NovaSure® system applies bipolar radiofrequency energy to thermally ablate the endometrial lining [3].

Limited data are available on histological changes following NovaSure® endometrial ablation. Available studies describe pronounced alterations, including stromal fibrosis, vascular changes, and a characteristic zonal pattern. These changes are described as being most prominent shortly after ablation, while diminishing over time [4, 5].

The incidence of atypical endometrial hyperplasia (AEH) and endometrial cancer (EC) in premenopausal patients with abnormal uterine bleeding (AUB) is substantially lower than in patients with postmenopausal bleeding, but still occurs in approximately 1.3% of cases [6]. NovaSure® endometrial ablation must not be used if there are indications of (pre-) malignant lesions in the endometrium. However, small or focal EC and AEH may remain undetected despite adequate examination, indicating the limited sensitivity of endometrial biopsy. Concordance between Pipelle biopsy and final hysterectomy specimens has been reported to be as low as 60%, particularly in patients without preprocedural clinical suspicion of malignancy [7]. To date, data are lacking on whether EC or AEH remain histologically detectable after NovaSure® ablation and whether diagnostic or therapeutic limitations may arise. This study aims to characterize the histological patterns following ablation in NovaSure® hysterectomy specimens. It will investigate whether residual atypia or carcinoma can be reliably identified and whether there are indications of potential diagnostic and therapeutic challenges.

## Methods

### Cohort and study design

A retrospective database analysis was conducted at our center between January 2020 and February 2025. Among more than 400 NovaSure® procedures, the pathological findings of curettage specimens obtained immediately before ablation were analyzed. AEH was detected in 8 patients, and EC cells were detected in 3 additional cases. In all cases, hysterectomy specimens from subsequent treatment were available at our center and were evaluated for histopathological characteristics. Patient and procedural characteristics of the 11 corresponding patients are presented in Table 1. The study was conducted in accordance with the approval of the Ethics Committee of Friedrich-Alexander University Erlangen-Nürnberg (approval number: 24-41-Br). This study presents a descriptive analysis of patient characteristics, treatment modalities, and histomorphological features.

**Table 1:** Baseline characteristics

Variable	Mean (SD), median (IQR) or number of participants (percentage %), *N* = 11	
Patient characteristics		
Age (years)	Mean (SD)	45.2 (5.3)
	Median (IQR)	45 (41, 50)
BMI (kg/m^2^)	Mean (SD)	33.8 (10.6)
	Median (IQR)	33.3 (23.5, 41.1)
Menopausal status	Premenopausal	11 (100)
Pre-existing conditions	Hematologic	1 (9.1)
	Cardiovascular	3 (27.3)
	Respiratory	2 (18.2)
	Thyroid dysfunction	1 (9.1)
	No Comorbidities	6 (54.5)
Gravidity (G)	G0	1 (9.1)
	G1	2 (18.2)
	G2	3 (27.3)
	≥ G3	5 (45.5)
Parity (P)	P0	1 (9.1)
	P1	2 (18.2)
	P2	4 (36.4)
	≥ P3	4 (36.4)
	≥ 1 SVD, no CS	5 (45.5)
	≥ 1 CS, no SVD	3 (27.3)
	Both CS and SVD	2 (18.2)
Prior uterine surgeries^1^	≥ 1 endometrial curettage	4 (36.4)
	None	7 (63.6)
Symptoms and clinical findings	Dysmenorrhea	5 (45.5)
	Anemia	4 (36.4)
	None	3 (27.3)
Previous therapy	OC	2 (18.2)
	None	9 (81.8)
Endometrial thickness (mm; US)	Mean	11.0 (5.1)
	Median (IQR)	12 (6, 15)
Time to hysterectomy after endometrial ablation (days)	EC, absolute values (*N* = 3)	28, 35, 35
	AEH, Mean (SD; *N* = 8)	48.5 (23.5)
	AEH, Median (IQR; *N* = 8)	43.5 (33.5, 59.5)
Procedural characteristics		
Concomitant procedures	Hysteroscopic myomectomy	1 (9.1)
	None	10 (90.9)
Working time (sec)	Mean (SD)	61.1 (28.6)
	Median (IQR)	49 (40, 99)
RF power (w)	Mean (SD)	135.9 (34.6)
	Median (IQR)	129 (106.5, 170)
Uterine length (cm)	Mean (SD)	6.2 (0.5)
	Median (IQR)	6.5 (6.0, 6.5)
Cavity width (cm)	Mean (SD)	3.9 (0.9)
	Median (IQR)	3.6 (3.1, 4.8)
Hysteroscopic findings	Uterine polyp	2 (18.2)

^1^excluding patients who had undergone cesarean section

*SVD* spontaneous vaginal delivery; *CS* cesarean section; *OC* oral contraceptives; US, preoperative ultrasound

### Pathological evaluation of specimens

During routine pathological workup, representative resection specimens were photographed. Paraffin-embedded blocks from all cases were selected, and corresponding H&E-stained sections were digitized using a Hamamatsu S210 slide scanner at 40 × magnification (0.1213 µm/pixel). Two pathologists with several years of experience (A.H. and C.M.) digitally reviewed all cases. Specific histologic alterations were subsequently discussed in joint review until a consensus diagnosis/classification was reached. Specifically, consensus values were obtained for estimated percentage of residual vital endometrium, quality of inflammatory pattern, as well as blood and lymphatic vessel alterations. Additionally, presence of residual atypia, necrosis, fibrotic and myxoid changes, as well as the zonation phenomenon described previously were assessed [4]. Representative image cutouts of the WSI scans were obtained to illustrate the respective histologic changes.

## Results

**Fig. 1:**
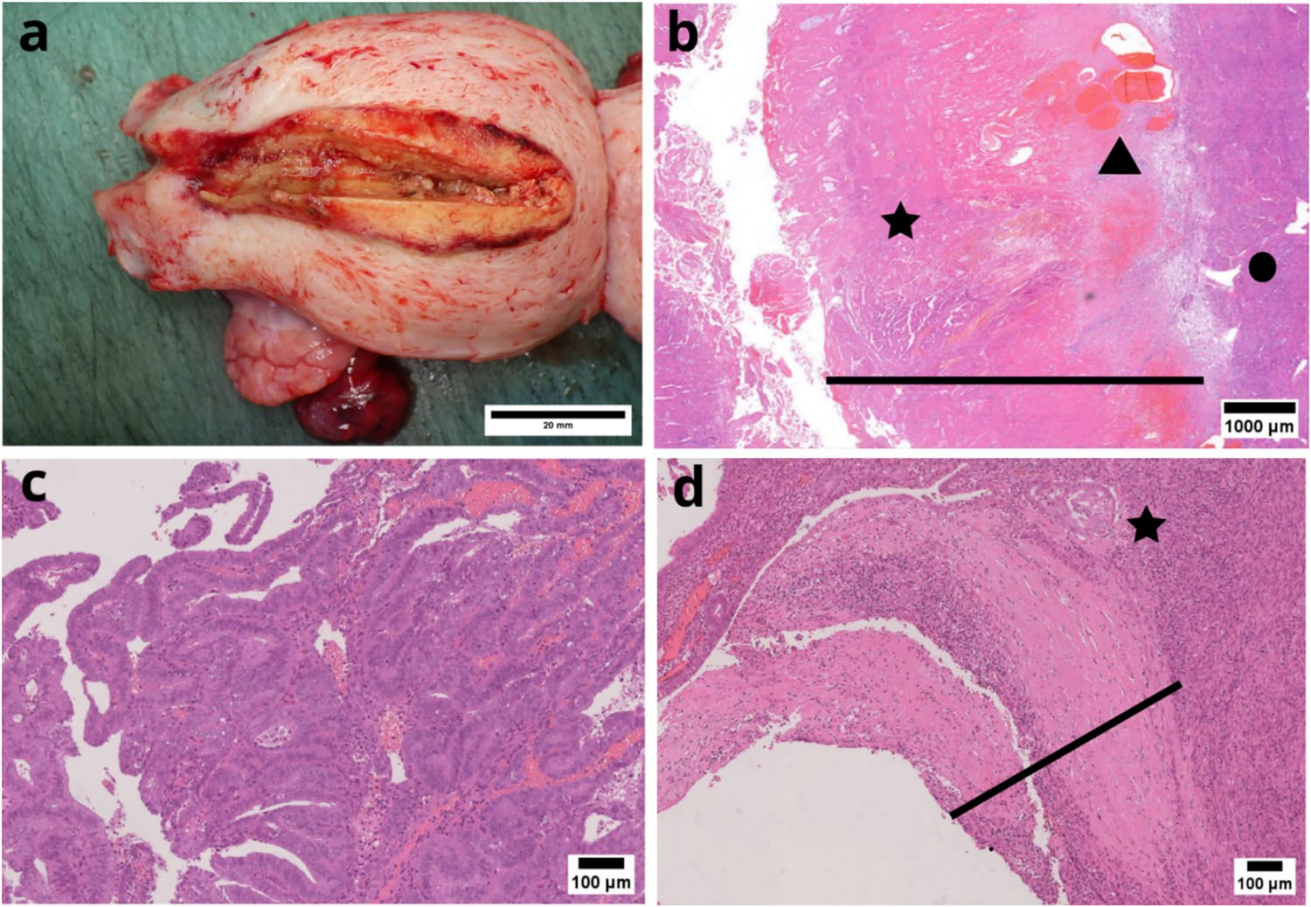
Panels A–C illustrate histological and macroscopic findings in the same patient with EC, in whom no residual carcinoma cells were detected in the hysterectomy specimen. Panel D shows a different patient with an initial diagnosis of AEH and residual atypia detected in the hysterectomy specimen. Macroscopical and microscopical findings: **a** macroscopical changes with necrotic endometrium in a patient with former EC. **b** Microscopical changes with superficial necrotic endometrium (line) and avital glandular silhouettes (star), vascular ectasia and hemorrhage in the mid-zone (triangle), and normal myometrium with lymphatic ectasia in the deeper zone (circle), HE. **c** Former well-differentiated EC, HE. **d** Residual atypical endometrioid glands (star) under subtotal avital endometrium (line), HE

**Fig. 2:**
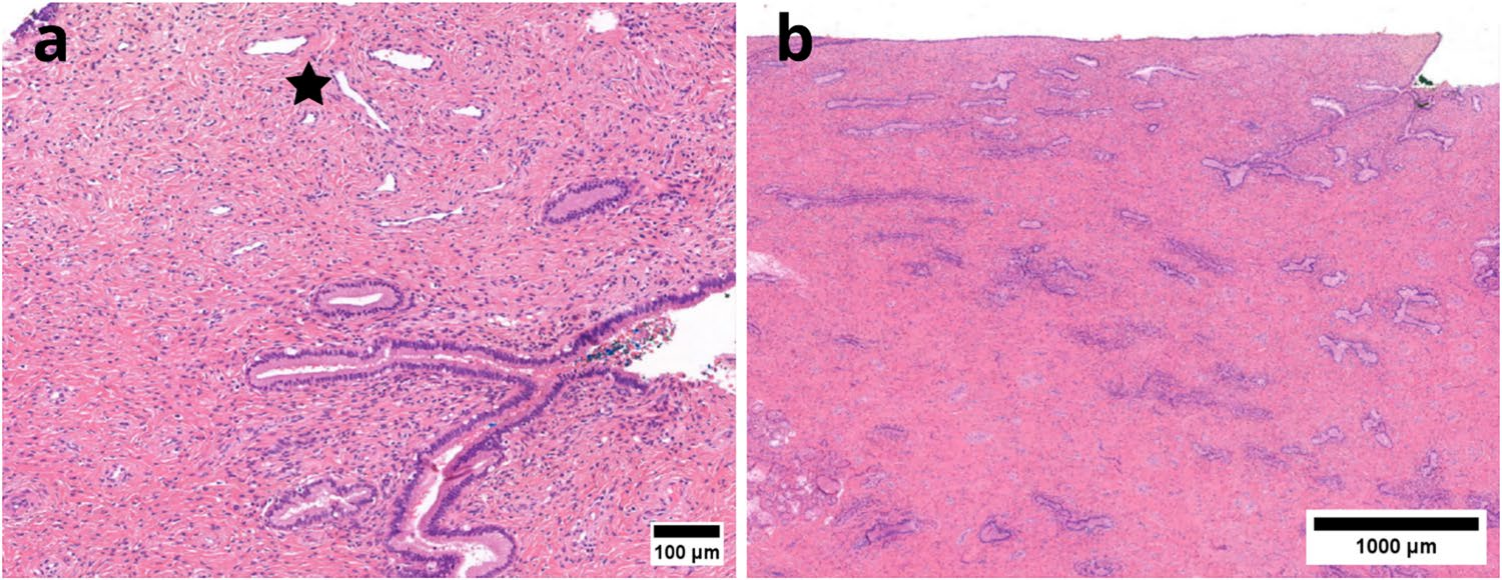
Histopathological findings in the endocervical region following NovaSure® endometrial ablation. **a** Endocervical area with no relevant change within the lymph vessels (star), HE. **b** Completely vital endocervical glands, HE

A total of 11 patients were included: 3 with EC and 8 with AEH. In all 3 EC cases, endometrioid endometrial carcinoma was diagnosed, with histopathological grade 1 (G1) tumors in two cases and a grade 2 (G2) tumor in one case. Molecular characterization, including immunohistochemical assessment of mismatch repair (MMR) proteins and p53 expression, was available in 2 of the 3 cases. Both analyzed cases demonstrated MMR proficiency (pMMR) and a wild-type p53 expression pattern. All patients with carcinoma underwent guideline-based oncological treatment, including hysterectomy combined with either sentinel lymph-node biopsy or pelvic lymphadenectomy. Sentinel lymph-node mapping after intracervical injection of indocyanine green (ICG) showed adequate intraoperative detection rates in all 3 cases. Postoperatively, all carcinoma patients were classified as FIGO stage IA, with no evidence of lymph-node involvement. All eight patients with AEH underwent hysterectomy with bilateral salpingectomy. Histopathological examination of the hysterectomy specimens revealed that 6 of the 8 patients with a preoperative diagnosis of AEH showed no residual atypia. Similarly, in all 3 patients with histologically confirmed EC, no remaining invasive lesions were detected in the final hysterectomy specimens. Overall, most cases showed only limited residual vital endometrium. Necrotic and fibrotic changes were common, as were ectatic and thrombotic blood vessels. Most cases showed a typical zonation consisting of a superficial necrosis zone inside the endometrium, a subendometrial hemorrhagic zone with ectatic blood vessels and extravasation of blood, and a deeper fibrotic zone with lightly ectatic lymph vessels, giving the endo- and myometrium a characteristic histological trizonal structure. An overview of the distribution of consensus histologic alterations is provided in Table 2 and illustrated in Fig. 1. Further histopathological evaluation of the endocervical region revealed no relevant alterations of the lymphatic vessels. The endocervical glands appeared completely viable, with preserved glandular architecture and no evidence of thermal damage or ablation-related changes. Representative histological findings are shown in Fig. 2.

**Table 2:** Histologic findings in hysterectomy specimens

Histologic Feature	Value	Number (percentage %), N = 11
Necrosis	Present	10 (90.9)
	Absent	1 (9.1)
Residual vital endometrium	Low (0–35%)	8 (72.7)
	Intermediate (35–75%)	1 (9.1)
	High (75–100%)	2 (18.2)
Residual atypia	Extensive	0
	Focal	2 (18.2)
	No	9 (81.8)
Fibrosis	Yes	8 (72.7)
	No	3 (27.3)
Zonation	Yes	7 (63.6)
	Yes (bilayered)	1 (9.1)
	No	3 (27.3)
Myxoid change	Yes	2 (18.2)
	Minimal	1 (9.1)
	No	8 (72.7)
Inflammatory infiltrate	Mixed	1 (9.1)
	Lymphocytic	7 (63.6)
	None	3 (27.3)
Blood vessel changes	Ectasia with thrombi	6 (54.5)
	Ectasia	4 (36.4)
	None	1 (9.1)
Lymphatic vessel changes	Normal	2 (18.2)
	Enlarged	9 (81.8)

## Discussion

Only a few studies have investigated the morphological features of hysterectomy specimens following endometrial ablation. Karpathiou et al. described time-dependent histological changes after thermal endometrial ablation, with hysterectomies performed early showing pronounced necrosis, fibrosis, vascular alterations, and a zonation effect. Specimens obtained at later time points demonstrated less prominent changes, and an almost normal endometrial lining; necrosis of myometrial origin and a high prevalence of adenomyosis were observed irrespective of the time interval [4]. In line with these observations, our cohort likewise exhibited high rates of necrosis, fibrosis, zonation, and alterations in vascular and lymphatic vessels. However, evaluating the time course of these changes was not possible, as all hysterectomies in our study were performed within a limited window of up to 96 days after endometrial ablation.

To date, data specifically characterizing hysterectomy specimens from patients with confirmed EC or AEH following endometrial ablation are lacking. In our cohort, only 2 of 11 cases showed focal residual atypia in the hysterectomy specimen, which is consistent with the overall low amount of residual endometrium, as in 73% of cases, there was little or no endometrial tissue remaining.

Histological confirmation prior to ablation is essential, as relevant premalignant or malignant lesions may be obscured by the procedure. This is particularly important as a considerable proportion of patients with biopsy-confirmed AEH also have EC in the subsequent hysterectomy specimen, with reported rates ranging from approximately 10% up to more than 50% [8–12]. Kurosawa et al. reported comparable rates, with concurrent EC detected in 41% of patients despite preoperative hysteroscopic inspection and total curettage, demonstrating that even combined hysteroscopic visualization and extensive endometrial sampling may miss focal malignant lesions or underestimate the extent of disease [13]. These findings are further supported by the study of Parsons et al., who investigated nearly 7000 patients undergoing hysterectomy for benign indications and identified 13 cases of occult EC (0.19%). Of these, 10 patients had undergone prior endometrial assessment by endometrial biopsy or dilation and curettage, and none had shown atypical hyperplasia or carcinoma preoperatively [14]. These findings support the need for thorough histologic evaluation prior to endometrial ablation and careful clinical treatment planning, while also underlining the inherent limitations of preoperative endometrial assessment.

**Fig. 3:**
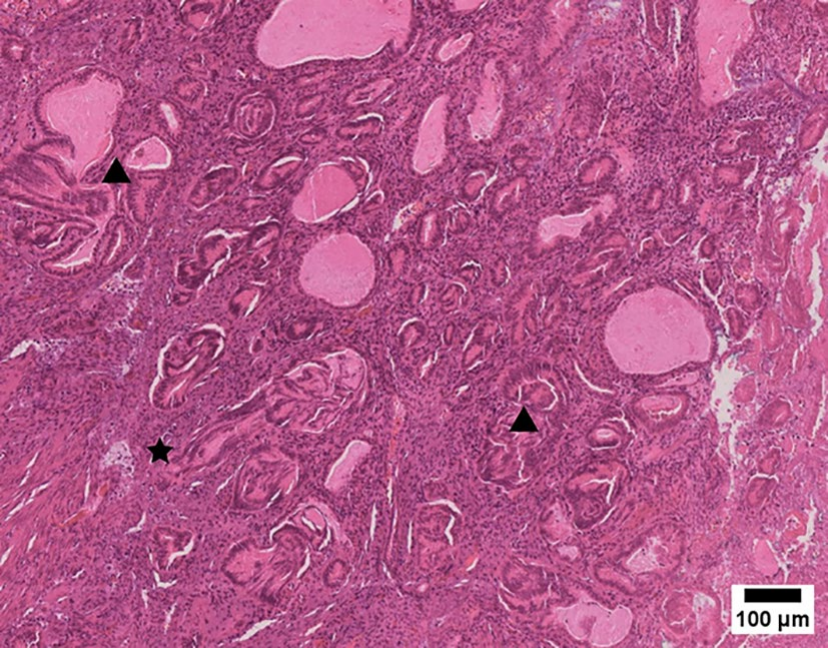
Thermally altered endometrial glands with cell nucleus crushing (star) and distortion of glandular architecture (triangle), HE

In our cohort, pronounced histological alterations of the lymph vessels and blood vessels attributable to the thermal effects of NovaSure® treatment were observed (Table 2), which are of immediate relevance for pathological assessment. However, there was no evidence that these features pose a significant risk of misinterpretation as malignant, as they are not usually located within the central necrotic zone and can be reliably distinguished from malignant glands by their flat endothelial lining and characteristic architectural features. The main diagnostic challenge for pathologists is distinguishing residual malignant cells from non-neoplastic cellular remnants within the necrotic ablation zone, where pronounced thermal and crush artifacts can markedly distort tissue morphology and thereby compromise histopathological interpretability. An illustrative example is shown in Fig. 3.

The absence of histologically detectable residual carcinoma should not be interpreted as a treatment effect of endometrial ablation**.** It may also be explained by complete removal during curettage, superficial tumor spread, or postablative sampling effects. It should also be noted that subsequent endometrial assessment may be compromised after prior thermal endometrial ablation. Both endometrial sampling and sonohysterography have been reported to fail more frequently in this patient group, thereby complicating further diagnostic evaluation [15].

Sentinel lymph-node biopsy is an established component of oncologic surgical treatment in endometrial cancer [16]. Sentinel tracer injection is performed intracervically and follows distribution along paracervical lymphatic pathways [17]. As NovaSure® endometrial ablation is confined to the uterine cavity and, consistent with our histomorphological findings, does not induce thermal alterations in the adjacent endocervical region, preserved cervical lymphatic architecture would be expected (Fig. 2). Adequate intraoperative detection of the sentinel lymph nodes was achieved in all 3 EC cases, and sentinel lymph-node biopsy was, therefore, performed in accordance with guideline-based surgical management. Systematic lymphadenectomy was carried out in cases with a specific clinical indication, such as intraoperatively suspicious lymph nodes. In this context, our findings provide no evidence of a limitation of the sentinel lymph-node mapping procedure following NovaSure® endometrial ablation.

## Conclusion

This study provides a descriptive analysis of clinical and histological parameters in a unique patient cohort. Pronounced histomorphological changes were observed after ablation, which are relevant for pathological assessment as they are relevant for delineating ablation-related changes and highlight diagnostic challenges. In particular, within the necrotic ablation zone, thermal and crush artifacts can impair histological interpretability. Preserved endocervical lymphatic structures suggest that sentinel lymph-node mapping based on intracervical tracer injection after NovaSure® ablation is not fundamentally impaired. However, these observations must be interpreted with caution, given the cohort’s descriptive nature and limited size. While the rarity of this clearly defined patient group and the detailed clinical–pathological correlation are significant strengths, the small sample size limits the generalizability. Accordingly, this study aims to provide a descriptive framework rather than therapeutic recommendations.

## Data Availability

The datasets generated and/or analyzed during the current study are not publicly available but are available from the corresponding author on reasonable request.
